# Prognostic value of baseline, interim and end-of-treatment ^18^F-FDG PET/CT parameters in extranodal natural killer/T-cell lymphoma: A meta-analysis

**DOI:** 10.1371/journal.pone.0194435

**Published:** 2018-03-20

**Authors:** Hongxi Wang, Guohua Shen, Chong Jiang, Li Li, Futao Cui, Rong Tian

**Affiliations:** Department of Nuclear Medicine, West China Hospital of Sichuan University, Chengdu, Sichuan Province, China; Northwestern University Feinberg School of Medicine, UNITED STATES

## Abstract

The prognostic value of ^18^F-fluorodeoxyglucose positron emission tomography-computed tomography (^18^F-FDG PET/CT) in extranodal natural killer/T-cell lymphoma (ENKTL) is currently controversial. Furthermore, whether the maximum standardized uptake value (SUV_max_), metabolic tumor volume (MTV), total lesion glycolysis (TLG) and Deauville 5-point scale (DS) acquired from PET/CT are predictors of prognosis in ENKTL remains unclear. The aim of this study was to explore the relationship between baseline, interim and end-of-treatment PET/CT (B-PET/CT, I-PET/CT and E-PET/CT) parameters and ENKTL prognosis.

**Methods:**

We searched the PubMed, EMBASE, Cochrane Library and Medline databases for eligible articles. SUV_max_, MTV, and TLG on B-PET/CT, DS on I-PET/CT and DS on E-PET/CT were regarded as efficacy data. Combined hazard ratios (HRs) for progression-free survival (PFS) and overall survival (OS) were estimated using RevMan 5.3 software.

**Results:**

Nine trials with a total of 535 ENKTL patients were included. SUV_max_, MTV and TLG on B-PET/CT were significantly associated with PFS with HRs of 2.78 (95%CI 1.54–5.03), 3.61 (95%CI 1.96–6.65) and 5.62 (95%CI 1.94–16.33), respectively, and with OS with HRs of 4.78 (95%CI 2.29–9.96), 3.20 (95%CI 1.55–6.60) and 7.76 (95%CI 1.79–33.58), respectively. For the DS on I-PET/CT, the HRs for PFS and OS were 5.15 (95%CI 2.71–9.80) and 5.80 (95%CI 2.28–14.73), respectively. Similarly, the DS on E-PET/CT was a significant predictor of PFS and OS with HRs of 3.65 (95%CI 2.13–6.26) and 3.32 (95%CI 1.79–6.15), respectively.

**Conclusion:**

Our results suggest that SUV_max_, MTV, TLG on B-PET/CT, DS on I-PET/CT and DS on E-PET/CT may be significant prognostic indicators for PFS and OS in ENKTL patients. Moreover, TLG tends to be superior to SUV_max_ and MTV on B-PET/CT for predicting survival of ENKTL patients. Therefore, response monitoring and prognostication assessments based on multiple PET/CT parameters should be considered in the management of ENKTL patients.

## Introduction

Extranodal natural killer/T-cell lymphoma (ENKTL) is a highly aggressive type of lymphoma with a median survival time of less than 12 months and with a remarkable geographical prevalence in Asia and South America [1–2]. No standard effective treatment currently exists, as ENKTL is refractory to chemotherapy and is associated with a high rate of therapeutic failure and poor prognosis [3–4]. Developing optimal approaches for the early identification of patients at high risk of progression or relapse is important in clinical management. The most common approaches include histopathological subtyping and use of the International Prognostic Index (IPI) [5] and the Korean Prognostic Index (KPI) [6]. Additionally, the immunophenotype and gene expression pattern can be prognostic factors. However, previous prognostic indicators based on the presented parameters have several drawbacks, including lack of consideration of the lymphoma response to treatment and insufficiency for accurately identifying patients with immunochemotherapy-refractory disease [7–8].

Currently, ^18^F-fluorodeoxyglucose (FDG) positron emission tomography-computed tomography (PET/CT) plays an important role in staging, response monitoring and prognostication assessments of lymphoma, especially Hodgkin lymphoma (HL) and diffuse large B-cell lymphoma (DLBCL) [9–11]. As a commonly used semi-quantitative parameter, the maximum standardized uptake value (SUV_max_) is a survival predictor that is associated with tumor aggressiveness [12–14]. Several recent studies have shown that quantitative metrics including metabolic tumor volume (MTV) and total lesion glycolysis (TLG) are reliable prognostic indicators with high sensitivity and reliability in DLBCL [9–10, 15]. Additionally, the five-point Deauville score (DS) has been recommended as a qualitative method for evaluating interim and end-of-treatment PET/CT results with good reproducibility and flexibility [16–17]. Although previous studies have evaluated the prognostic value of these PET/CT parameters for various types of lymphoma, similar studies of ENKTL have been limited by sample size. Moreover, previous findings remain controversial due to heterogenous patient enrollment, various imaging conditions, different cut-off values for survival predictions, and undefined treatment protocols. Therefore, the purpose of this meta-analysis was to evaluate the prognostic value of multiple PET/CT parameters including SUV_max_, MTV, TLG and DS in ENKTL at three different time points: baseline, interim and end of treatment.

## Materials and methods

### Literature search

A systematic digital literature search was performed to identify studies that assessed the value of PET/CT parameters in evaluating the prognosis of ENKTL patients. The PubMed, EMBASE, Cochrane Library and Medline databases were searched for English language articles with the following keywords: (“positron emission tomography” or “positron emission tomography/computed tomography”), (“PET/CT” or “PET”) and (“Lymphoma, Extranodal NK-T-Cell”, “NK/T-cell lymphoma” or “ENKTL”). No start date limit was used, the search was last updated on January, 2018. Additionally, reference lists from the included studies were also searched.

### Inclusion and exclusion criteria

The criteria for including studies in the meta-analysis were as follows: (i) prospective or retrospective studies that used at least one of three PET/CT scans, namely, B-PET/CT, I-PET/CT and E-PET/CT, to assess therapeutic responses and to predict ENKTL patient survival; (ii) studies that included more than 10 patients with histologically confirmed ENKTL; (iii) studies containing survival data from which the hazard ratio (HR) was extractable, reporting at least one form of survival data progression-free survival (PFS) or overall survival (OS), or providing sufficient data for indirect extraction; (iv) when the data were presented in more than one study, the studies with the most complete or most recently published data were included; (v) and studies written only in English. The exclusion criteria were as follows: (i) studies focusing on other diseases or lymphoma subtypes; (ii) studies that evaluated the diagnostic or staging value rather than the prognostic value of PET/CT in ENKTL; (iii) studies in which ENKTL patients could not be separated from patients with other lymphoma subtypes; (iv) studies lacking survival data or studies in which the data could not be indirectly extracted; (v) or studies lacking original patient data, such as case reports, conference proceedings, commentaries and reviews. All articles were identified by two reviewers using the aforementioned criteria.

### Data extraction and quality assessment

Data were extracted by two reviewers with differences resolved by consensus. Extracted data included the following: study characteristics, including title, author, and publication time; patient characteristics, including age, gender, Ann Arbor stage, IPI score, treatment regimen, and follow-up time; imaging techniques, including the imaging system, interval time between FDG administration and scanning, FDG dose, imaging interpreters, prognostic parameters and cut off value; and survival data, including PFS and OS with HRs with 95% confidence intervals (95% CIs). SUVmax was quantified as the measure of the greatest amount of FDG uptake in a region of interest normalized to body weight. MTV was determined from the attenuation-corrected PET data using the software. TLG was calculated as the sum of the individual MTVs multiplied by the SUVmean of every lesion. DS was defined as follows: 1, no uptake; 2, uptake ≤the mediastinum; 3, uptake > the mediastinum but ≤ the liver; 4, uptake that was moderately greater than the liver; 5, uptake markedly greater than the liver and/or the presence of new lesions; and X, new areas of uptake unlikely to be related to lymphoma [16–17].

The quality of the studies was evaluated independently by two reviewers. The Quality in Prognosis Studies (QUIPS) tool [18] was used to estimate the quality of the included studies. Using this tool, the validity and bias of the studies of prognostic factors were evaluated for the following six domains: study participation, study attrition, prognostic factor measurement, outcome measurement, study confounding, and statistical analysis and reporting. Each domain was rated as having a high, moderate, or low risk of bias considering the prompting items.

### Statistical analysis

The HR was used for PFS and OS in the present systematic review. When extracting the HR, we attempted to use the HR from the multivariate Cox analysis reported within the text of the report. When a study did not provide this information, we used the HR from the univariate Cox analysis instead. If the trial did not provide the HR directly, we attempted to indirectly extract it from the Kaplan-Meier survival curve as previously described by Parmar et al. [19] and Tierney et al. [20]. Statistical heterogeneity was measured using the chi-squared Q test and the I^2^ statistic. We considered heterogeneity to be present at P<0.05 or/and I^2^>50%. A fixed effects model was used for the meta-analysis when heterogeneity was not significant, and a random effects model was used if heterogeneity was significant. The analyses described above were conducted by Review Manager (RevMan, version 5.3; The Nordic Cochrane Center, The Cochrane Collaboration). Begg’s test and Egger’s test were used to assess publication bias by STATA Version 14.0 (Stata Corp LP, College Station, TX 77845, USA), and p<0.05 was considered statistically significant.

## Results

### Literature search and selection of studies

**Fig 1:**
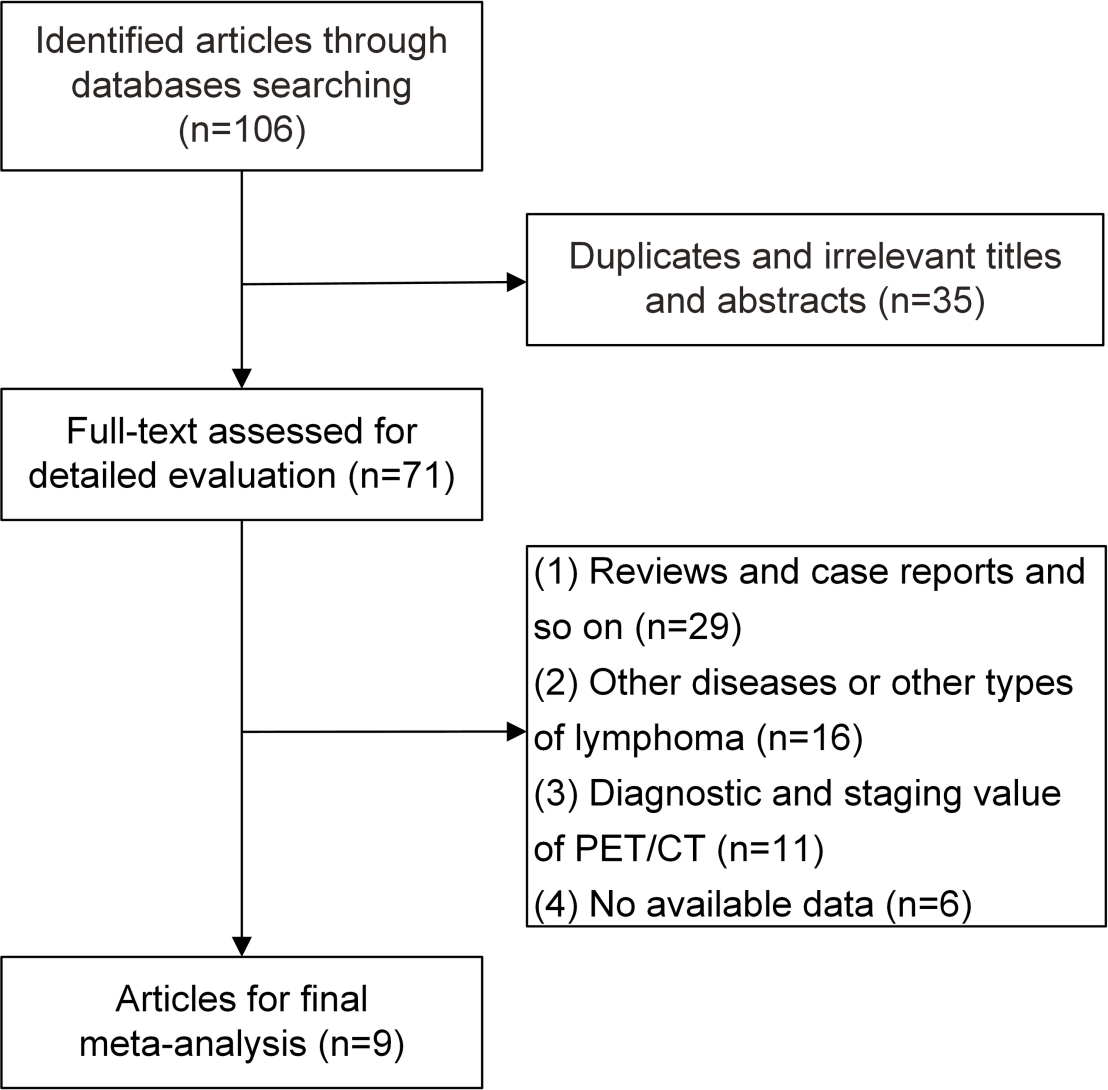
Flow diagram of study selection.

The search strategy was used to screen 106 records for inclusion. After discarding duplicate and irrelevant articles by reading the titles and abstracts, we reviewed 71 studies in detail. Among these articles, 62 studies were excluded for the following reasons: a lack of original data, such as case reports, letters, conference proceedings, commentaries and reviews (n = 29); a focus on other diseases or lymphoma types (n = 16); an aim toward revealing the diagnostic or staging value of PET/CT in ENKTL (n = 11); or insufficient data to calculate PFS, OS and the HRs for each (n = 6). Ultimately, nine studies [21–29] were selected for the meta-analysis (Fig 1).

### Study design characteristics and methodological quality

The principal characteristics of the nine studies are described in Table 1. All studies, including three prospective studies [24–25] and six retrospective studies [21–23, 27–29], were published between 2013 and 2017 and were performed at Asian institutions. The median follow-up time ranged from 19 to 45 months. Five studies [22–23, 27–29] focused on the prognostic value of B-PET/CT, three studies [22, 24–25] addressed the prognostic value of I-PET/CT performed during the 4 weeks after initial treatment, and three studies [21–22, 26] revealed the prognostic value of E-PET/CT performed after the final treatment with an interval of 3–4 weeks. Liang et al. [23] used WB3SUVmax, which represents the whole body SUVmax of 3 nodal regions (superior diaphragm, inferior diaphragm and spleen) and 10 extranodal regions (upper aero-digestive tract, skin/subcutaneous tissues, central nervous system and spinal cord, lung, myocardium, bone and bone marrow, bowel, kidney and adrenals, liver and testis) as prognostic factors. Although WB3SUVmax does not represent the total metabolic activity of lymphoma sites, it represents the sum of the highest metabolic rates of all nodal and extranodal lesions and its values are generally concordant compared with SUVmax. Due to the limited number of included studies, we classified WB3SUVmax as SUVmax for the meta-analysis. In the study by Jim et al. [24], all patients were treated with induction therapy combined with chemotherapy or CCRT and then received PET/CT assessments before autologous stem cell transplantation. Due to the limited number of included studies, we categorized these data as interim PET/CT scans for the analysis. The PET/CT imaging and interpretation methods are shown in Table 2.

**Table 1:** Characteristics of the nine included studies and their corresponding patients.

Study reference	Country	Study design	Sample (M/F)	Age^a^	AA stage (I-II/III-IV)	IPI (0-1/2-5)	LDH (normal/increased)	B symptoms (absent/present)	Chemotherapy regimen	Follow-up time^b^
**Jiang et al, 2017 [21]**	China	R	59 (37/22)	41 (13–63)	53/6	NR	37/22	28/31	VDLP, cisplatin	25 (8–58)
**Chang et al, 2017 [22]**	China	R	52 (31/21)	40.5 (15–72)	34/18	38/14	29/23	31/21	DDGP, modified SMILE	19 (5–55)
**Liang et al, 2016 [23]**	China	R	54 (37/17)	46 (14–85)	27/27	23/31	26/28	31/23	L/PEMD	45 (20–120)
**Lim et al, 2016 [24]**	South Korea	P	27 (19/8)	44 (19–64)	13/14	14/13	9/18	10/17	SMILE, VIDL, VIPD, GDP	36.9 (1.6–75.4)
**Jiang et al, 2015 [25]**	China	P	60 (36/24)	40.8 (18–68)	44/16	45/15	45/15	42/18	LVP	23.5 (9–36)
**Kim et al, 2015 [26]**	South Korea	R	102 (66/36)	48 (17–75)	68/34	64/38	55/47	65/37	cisplatin, VIPD, VIDL, SMILE	47.2 (30.0–65.5)
**Kim et al, 2013 [27]**	South Korea	R	20 (13/7)	55 (19–85)	15/5	12/8	11/9	14/6	CHOP/CHOP-like, EPOCH	26.3
**Bai et al, 2013 [28]**	China	R	81 (58/23)	41 (14–74)	68/13	64/17	62/19	45/36	Anthracycline /L-ASP	25.0 (2.0–99.0)
**Song et al, 2013 [29]**	South Korea	R	80 (47/33)	59 (23–74)	51/29	56/24	59/21	67/13	Doxorubicin, CHOP or another regimen	35.6 (10.2–72.5)

^a^ Data are presented as the means and ranges of the patients’ ages in years.

^b^Data are presented as the means and ranges of follow-up times in months.

M, male; F, female; AA stage, Ann Arbor stage; IPI, International Prognostic Index; RT, radiotherapy; R, retrospective; P, prospective; NR, not reported.

**Table 2:** PET/CT imaging and interpretation methods.

Study reference	Post injection interval (min)	FDG dose (MBq/kg)	parameters	Cut-off values			Interpreters	Time interval before I-PET	Time interval before E-PET
				SUVmax	MTV (mL)	TLG (g)			
**Jiang et al, 2017 [21]**	60	5.18	E-PET/CT DS				2 physicians specializing in nuclear medicine	During the 4 weeks after initial treatment	3 weeks after the end of treatment
**Chang et al, 2017 [22]**	60±15	3.7–4.4	B-PET/CT SUV_max_, MTV and TLG	15.1	16.1	44.7	2 experienced nuclear medicine physicians	Median 19.0 days (after 2–4 cycles of chemotherapy)	Median 31.5 days
**Liang et al, 2016 [23]**	60	3.70–5.55	B-PET/CT SUV_max_	15.8			NR	NR	NR
**Lim et al, 2016 [24]**	NR	NR	I-PET/CT DS				1 nuclear medicine physician	NR	NR
**Jiang et al, 2015 [25]**	60	5.18	I-PET/CT DS				2 physicians specializing in nuclear medicine	3 weeks (after 2 cycles of therapy)	NR
**Kim et al, 2015 [26]**	60±10	5.5	E-PET/CT DS				1 experienced nuclear medicine physician	NR	At least 4 weeks after treatment
**Kim et al, 2013 [27]**	60	8.1	B-PET/CT SUV_max_, MTV and TLG	8.1	14.4	52.7	NR	NR	NR
**Bai et al, 2013 [28]**	60	4.4	B-PET/CT SUV_max_	15			2 experienced nuclear medicine physicians	NR	NR
**Song et al, 2013 [29]**	NR	NR	B-PET/CT MTV		35.2		1 nuclear medicine expert	NR	NR

NR, not reported.

The methodological quality of the nine studies was assessed using the QUIPS tool. Generally, the included studies were of moderate quality. In the domain of prognostic factor measurements, there was a moderate risk of bias in two studies (24, 29) because they did not reveal detailed information about the imaging acquisition. All studies were judged to be at moderate risk of bias in the domain of outcome measurements because they did not report whether a histological biopsy was used to confirm treatment failure or relapse. Due to clinical limitations associated with this disease, a biopsy of every lesion suspected of recurrence or progression is impossible. Finally, there was a moderate risk of bias due to study confounding in six studies (22, 24, 26–29) because these studies included patients treated with heterogeneous treatment regimens. The quality assessment of the included studies according to the six QUIPS domains is displayed in Table 3.

**Table 3:** Quality assessment of the included studies.

Study reference	Study participation	Study attrition	Prognostic factor measurement	Outcome measure	Study confounding	Statistical analysis
**Jiang et al, 2017 [21]**	L	L	L	M	L	L
**Chang et al, 2017 [22]**	L	L	L	M	M	L
**Liang et al, 2016 [23]**	L	L	L	M	L	L
**Lim et al, 2016 [24]**	L	L	M	M	M	L
**Jiang et al, 2015 [25]**	L	L	L	M	L	L
**Kim et al, 2015 [26]**	L	L	L	M	M	L
**Kim et al, 2013 [27]**	L	L	L	M	M	L
**Bai et al, 2013 [28]**	L	L	L	M	M	L
**Song et al, 2013 [29]**	L	L	M	M	M	L

L, low risk; M, moderate risk; H, high risk.

### Meta-analysis

**Fig 2:**
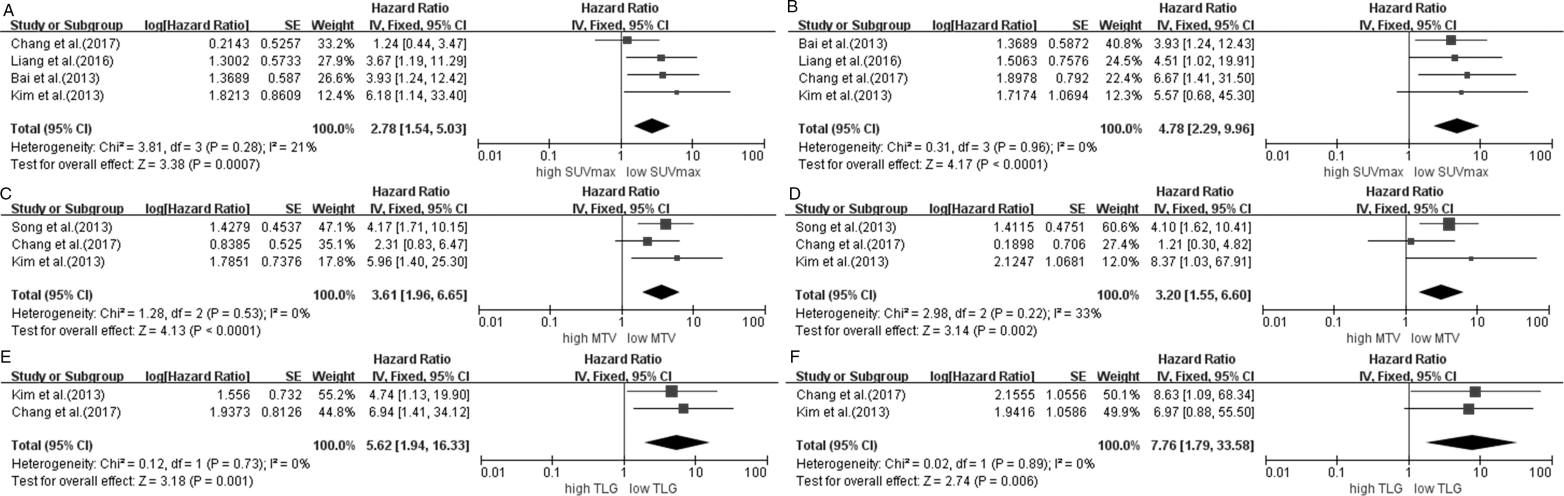
Forest plots of HR for PFS and OS with SUV_max_ (A, PFS; B, OS), MTV (C, PFS; D, OS) and TLG (E, PFS; F, OS) on B-PET/CT.

Six studies [22–23, 27–29] revealed the value of B-PET/CT in evaluating the prognosis for ENKTL. SUV_max_ [22–23, 27–28], MTV [22, 27, 29] and TLG [22, 27] were used to predict PFS and OS. For SUV_max_, the HRs for PFS and OS were 2.78 (95% CI 1.54–5.03, p = 0.0007; χ^2^ = 3.81, P = 0.28, I^2^ = 21%) (Fig 2A) and 4.78 (95% CI 2.29–9.96, p<0.0001; χ^2^ = 0.31, P = 0.96, I^2^ = 0%) (Fig 2B), respectively. Furthermore, there was an association between a high MTV value and poor PFS and OS with HRs of 3.61 (95% CI 1.96–6.65, p<0.0001; χ^2^ = 1.28, P = 0.53, I^2^ = 0%) (Fig 2C) for PFS and 3.20 (95% CI 1.55–6.60, p = 0.002; χ^2^ = 2.98, P = 0.22, I^2^ = 33%) (Fig 2D) for OS. For TLG, the HRs for PFS and OS were 5.62 (95% CI 1.94–16.33, p = 0.001; χ^2^ = 0.12, P = 0.73, I^2^ = 0%) (Fig 2E) and 7.76 (95% CI 1.79–33.58, p = 0.006; χ^2^ = 0.02, P = 0.89, I^2^ = 0%) (Fig 2F), respectively. Taken together, the SUV_max_, MTV and TLG of B-PET/CT were significant predictors for PFS and OS in ENKTL patients.

**Fig 3:**
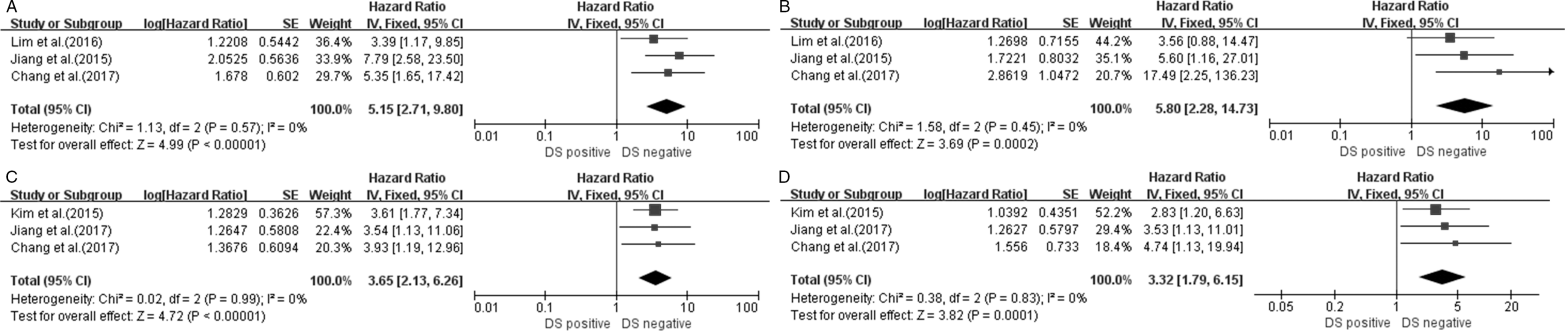
Forest plots of HR for PFS and OS with DS (A, PFS; B, OS) of I-PET/CT and DS (C, PFS; D, OS) on E-PET/CT.

Regarding the I-PET/CT parameters, three studies [22, 24–25] using DS were available, with HRs for PFS and OS of 5.15 (95% CI 2.71–9.80, p<0.00001; χ^2^ = 1.13, P = 0.57, I^2^ = 0%) (Fig 3A) and 5.80 (95% CI 2.28–14.73, p = 0.0002; χ^2^ = 1.58, P = 0.45, I^2^ = 0%) (Fig 3B), respectively, indicating a significant association between a positive DS in I-PET/CT and poor PFS and OS. Three studies [21–22, 26] were available regarding the DS on E-PET/CT, with HRs for PFS and OS of 3.65 (95% CI 2.13–6.26, p<0.00001; χ^2^ = 0.02, P = 0.99, I^2^ = 0%) (Fig 3C) and 3.32 (95% CI 1.79–6.15, p = 0.0001; χ^2^ = 0.38, P = 0.83, I^2^ = 0%) (Fig 3D), respectively, suggesting that a positive DS in E-PET/CT predicted worse survival.

ROC curves were used to evaluate cut-off values in all included studies. All pooled data were statistically homogeneous. In evaluating publication bias, the results of Egger’s test and Begg's test were both insignificant (p>0.05), indicating no publication bias for the HRs.

## Discussion

As a rare cancer type with a relatively high incidence in Asia and South America, ENKTL is diagnostically considered in significant proportions of patients undergoing disease progression or relapse after a good initial treatment response, especially when lesions are disseminated [7]. Zhou X et al. [30] performed a meta-analysis and systematic review to assess the role of 18F-FDG-PET/CT in the diagnosis and staging of NK/T-cell lymphoma in 135 patients from 6 studies, and results indicated that PET/CT represented a valuable diagnostic and staging tool for ENKTL. Further research is necessary to identify which prognostic indicators are the most suitable and reliable in the management of ENKTL patients and to determine how to optimize the use of prognostic indicators to identify individuals with poor prognoses. The current meta-analysis assessed the prognostic value of PET/CT at three different time points with four parameters comprising SUV_max_, MTV, TLG, and DS in ENKTL. Based on a statistical analysis of 535 ENKTL patients, our meta-analysis demonstrated that SUV_max_, MTV, TLG of B-PET/CT, DS on I-PET/CT and DS on E-PET/CT were predictors for PFS and OS in ENKTL patients, indicating that PET/CT can be used as a metabolic imaging approach to monitor treatment responses and to assess the prognosis of ENKTL patients in clinical practice.

Several studies have shown that baseline SUV_max_ can be a predictor of the treatment response and survival rate in ENKTL, which is consistent with our meta-analysis results [31–32]. Chang et al. [22] reported that a high SUV_max_ was an independent predictor of OS but not PFS. Conversely, Kim et al. [27] reported that a high B-PET SUV_max_ was a significant predictor of PFS but not OS. Whereas in the study of Kim et al. [27], the sample size was relatively small, and patients received CHOP/CHOP-like or EPOCH regimens, which may have led to disputable results regarding prognosis. Due to multidrug resistance in ENKTL, anthracycline-based chemotherapy (e.g., CHOP or CHOP-like regimens) is ineffective and has been replaced by an array of more effective non-anthracycline regimens [7].

For B-PET/CT, TLG tends to be superior to SUV_max_ and MTV in predicting survival. The reason might be that TLG is an ideal metabolic parameter that combines the SUV mean and MTV to combine assessments of tumor volume and metabolism. Our results are consistent with previous studies that showed the prognostic value of the PET/CT volume parameters for revealing the whole body metabolic tumor burden. Based on their cohort study of 103 DLBCL patients, Ceriani et al. [10] concluded that TLG was the most powerful predictor on baseline PET/CT. Mikhaeel et al. [15] found that the baseline PET MTV and TLG were the most prognostic quantitative measures and were superior to IPI and DS. In their study, Zhou et al. [9] found that tumor metabolic volume parameters were helpful in the management of DLBCL and that TLG was an especially striking predictor. Moreover, Chang et al. [22] reported that the negative predictive values (NPVs) of baseline SUV_max_, MTV and TLG for PFS and OS were higher than the positive predictive values (PPVs) in 47 ENKTL patients. Notably, the NPVs of TLG in predicting PFS and OS were 90.5% and 95.2%, respectively. Thus, negative results may have a stronger relationship with good PFS and OS than positive results have with poor PFS and OS.

However, our meta-analysis revealed that MTV was not superior to SUV_max_ regarding the combined HRs of both PFS and OS, and several reasons may explain this discrepancy. First, the three included studies [22, 27, 29] regarding MTV had too few patients to be conclusive. Second, MTV represents the size of tumor tissues that exhibit active ^18^F-FDG uptake, the MTV measurements are not reliable, and reproducibility, especially for multiple, disseminated, and extensive lesions, is poor [33]. Moreover, a standard method for estimating MTV thresholds has not been defined [34–36]. Chang et al. [22] estimated an MTV threshold based on 40% of the SUV_max_. Song et al. [29] and Kim et al. [27] used a fixed threshold with SUV_max_ values of 2.5 and 3.0, respectively. A fixed threshold may lack reproducibility due to the variability caused by biological and technological factors, whereas using a proportion of the SUV_max_ as a threshold may lead to misestimation of the calculated tumor volume in cases of heterogeneous or low uptake. Kanoun et al. [37] evaluated the impact of the differences in methodological calculations on MTV values in HL and found that MTV values were significantly affected by calculation methods; however, different MTV values were associated with prognosis. Several studies have reported that the adoption of an individualized thresholds based on the liver background can reduce the impact of different scanning techniques and patient factors in DLBCL and solid tumors [9, 38]. Thus, a standard measuring method for MTV is needed for more accurate assessments of ENKTL patients.

Additionally, our study suggests that positive DS on I-PET/CT has a significant association with reduced PFS and OS in ENKTL, and the results of our analysis based on DS on E-PET/CT are consistent with those of previous studies. In a study of 24 patients with newly diagnosed or relapsed histologically confirmed ENKTL, Khong et al. [32] concluded that mid-treatment PET/CT was a valuable tool for early treatment response assessments. In a cohort of 88 T/NK-cell lymphoma patients including 26 ENKTL patients, both interim and post-therapy PET/CT scans were independent predictors of PFS and OS [39]. I-PET/CT presumably plays a critical role in two aspects of clinical management of lymphoma by allowing evaluation of the therapeutic response and prediction of prognosis. Due to the rarity of ENKTL and lack of a current unified treatment strategy for ENKTL, data addressing issues such as whether altering therapy based on I-PET/CT results can improve patient survival and whether DS as a metric of I-PET/CT can accurately distinguish high-risk patients from low-risk patients in ENKTL are lacking. Thus, a conclusion on the practical usefulness of I-PET/CT in ENKTL cannot be clearly drawn.

Previous studies have confirmed that the DS, where values of 1–3 are defined as negative results and 4–5 are defined as positive results on I-PET/CT, can better predict PFS and OS, with good concordance among reviewers and inter-reader reliability in HL and NHL [40–42]. Jiang et al. [25] evaluated the prognostic value of I-PET/CT using the DS and found that NPVs were better than PPVs in predicting PFS and OS, with NPVs of 87.5% and 96.9%, respectively, and PPVs of 60.7% and 39.3%, respectively. The authors concluded that negative results may have a stronger relationship with good PFS and OS than positive results have with poor PFS and OS. However, Lim et al. [24] found that DS associated with EBV DNA has significant prognostic value in ENKTL patients who undergo autologous stem cell transplantation and that defining values of 3–5 as positive results can better differentiate survival. These data were similar to a study by Kim et al. [26], in which a new risk stratification method was suggested based on DS and EBV DNA at the end of treatment. Khong et al. [32] revealed that scores of 1–2 at the end of treatment may represent a complete metabolic response; the scores are equal to an evaluation using IHPC and may be more appropriate at the end of treatment to increase the NPV of PET. The Lugano classification suggests that a score of 3 generally indicates a good prognosis with standard treatment, especially at the interim scan. However, if de-escalation is investigated, it may be preferable to consider a score of 3 as an inadequate response to avoid undertreatment [17]. Therefore, a score of 3 should be carefully interpreted depending on the timing of the assessment, the clinical context, and the treatment choice. Moreover, the risk of inter-observer subjectivity in the interpretation has not been eliminated, especially for small or slightly enhanced lesions in the background with changes in physiological uptake [43]. In one report, 22% of the cases had to be discussed due to discrepant DS assessments between the two independent interpreters [44]. The combined assessment is presumably a better predictor of survival outcomes than the single-parameter assessment, thus optimizing this assessment metric and reducing inter-observer differences are important. Cai QQ et al. [45] proposed a prognostic model including fasting blood glucose, total protein, and KPI for ENKTL, and the results demonstrated that the model could distribute patients into different risk groups with better prognostic discrimination than KPI alone. Lee J et al. [46] used a prognostic model that included B symptoms, stage, lactate dehydrogenase (LDH) level, and regional lymph nodes. Kim et al. [47] created new prognostic models, PINK and PINK-E, that were performed after patients received non-anthracycline-based treatment and considered age, stage, distant lymph-node involvement, non-nasal type disease, and Epstein-Barr virus DNA to guide risk-adapted treatment. According to Ya Jun Li et al. [48], the Glasgow Prognostic Score (GPS), a cumulative prognostic score based on CRP and albumin levels, is an independent predictor of survival outcomes in low-risk groups of patients with ENKTL and is superior to IPI, PIT, and KPI. Few studies have explored prognostic models that consider PET/CT scan results in ENKTL. Our meta-analysis shows the significant prognostic values of PET/CT in ENKTL and that the response assessment and survival prediction based on multiple PET/CT parameters and clinical characteristics should be considered in the management of ENKTL patients.

The current meta-analysis has several limitations. First, only published English language articles were included, and all studies were from Asia. Second, the included studies enrolled relatively small numbers of subjects, and we analyzed a total of 535 ENKTL patients in this meta-analysis. Furthermore, the included studies were mostly retrospective, and the quality assessment was moderate. Registrations on Cochrane and PROSPERO are not available for this meta-analysis. These methodological study limitations contribute to deficiencies associated with the current study. Fourth, there were varying inclusion and exclusion criteria for patient enrollment among the included studies. According to Jiang et al. [21, 25], patients were excluded if they were diagnosed with primary cutaneous T/natural killer-cell lymphomas or anaplastic lymphoma kinase-positive anaplastic large cell lymphoma. In the cohorts of several included studies [21–22, 25], patients with central nervous system involvement were also excluded. Obviously, the treatment effect and prognosis may vary with histologic subtype or disease involvement. Finally, the minimum follow-up time was 19 months in the included studies. However, most relapses take longer to occur. If a longer follow-up time was used, the disease relapse rate would be higher, and the prognostic performance would be different.

## Conclusion

The current evidence demonstrates that SUV_max_, MTV, TLG of B-PET/CT, DS on I-PET/CT and DS on E-PET/CT may be significant prognostic indicators for PFS and OS in ENKTL. Moreover, for B-PET/CT, TLG tends to be superior to SUV_max_ and MTV in predicting the survival of ENKTL patients. Taken together, PET/CT assessments with multiple parameters may have better prognostic performance for determining ENKTL progression in patients. However, future large-scale prospective studies are needed to confirm the prognostic value of the multiple PET/CT parameters in ENKTL.

## Supporting information

S1 Checklist PRISMA checklist. (DOCX)

S1 Appendix Search strategy. (DOCX)

S1 Table Details of the survival extraction. (XLSX)
