# Supplementary material for: Prognostic value of baseline, interim and end-of-treatment 18F-FDG PET/CT parameters in extranodal natural killer/T-cell lymphoma: A meta-analysis
Source: PLoS One. 2018 Mar 20;13(3):e0194435. doi: 10.1371/journal.pone.0194435 (PMC5860776; doi:10.1371/journal.pone.0194435)
Supplement: S1 Checklist — (DOCX) [file pone.0194435.s001.docx]

| **Section/topic** | **#** | **Checklist item** | **Reported on page #** |
| --- | --- | --- | --- |
| **TITLE** | | |  |
| Title | 1 | Prognostic value of baseline, interim and end-of-treatment 18F-FDG PET/CT parameters in extranodal natural killer/T-cell lymphoma: A meta-analysis |  |
| **ABSTRACT** | | |  |
| Structured summary | 2 | The prognostic value of 18F-ﬂuorodeoxyglucose positron emission tomography-computed tomography (18F-FDG PET/CT) in extranodal natural killer/T-cell lymphoma (ENKTL) is currently controversial. Furthermore, whether the maximum standardized uptake value (SUVmax), metabolic tumor volume (MTV), total lesion glycolysis (TLG) and Deauville 5-point scale (DS) acquired from PET/CT are predictors of prognosis in ENKTL remains unclear. The aim of this study was to explore the relationship between baseline, interim and end-of-treatment PET/CT (B-PET/CT, I-PET/CT and E-PET/CT) parameters and ENKTL prognosis. Methods: We searched the PubMed, EMBASE, Cochrane Library and Medline databases for eligible articles. SUVmax, MTV, and TLG on B-PET/CT, DS on I-PET/CT and DS on E-PET/CT were regarded as efficacy data. Combined hazard ratios (HRs) for progression-free survival (PFS) and overall survival (OS) were estimated using RevMan 5.3 software. Results: Nine trials with a total of 535 ENKTL patients were included. SUVmax, MTV and TLG on B-PET/CT were significantly associated with PFS with HRs of 2.78 (95%CI 1.54-5.03), 3.61 (95%CI 1.96-6.65) and 5.62 (95%CI 1.94-16.33), respectively, and with OS with HRs of 4.78 (95%CI 2.29-9.96), 3.20 (95%CI 1.55-6.60) and 7.76 (95%CI 1.79-33.58), respectively. For the DS on I-PET/CT, the HRs for PFS and OS were 5.15 (95%CI 2.71-9.80) and 5.80 (95%CI 2.28-14.73), respectively. Similarly, the DS on E-PET/CT was a significant predictor of PFS and OS with HRs of 3.65 (95%CI 2.13-6.26) and 3.32 (95%CI 1.79-6.15), respectively. Conclusion: Our results suggest that SUVmax, MTV, TLG on B-PET/CT, DS on I-PET/CT and DS on E-PET/CT may be significant prognostic indicators for PFS and OS in ENKTL patients. Moreover, TLG tends to be superior to SUVmax and MTV on B-PET/CT for predicting survival of ENKTL patients. Therefore, response monitoring and prognostication assessments based on multiple PET/CT parameters should be considered in the management of ENKTL patients.  Key Words: Extranodal natural killer/T-cell lymphoma (ENKTL); Positron emission tomography-computed tomography (PET/CT); Progression-free survival (PFS); Overall survival (OS); Meta-analysis |  |
| **INTRODUCTION** | | |  |
| Rationale | 3 | Extranodal natural killer/T-cell lymphoma (ENKTL) is a highly aggressive type of lymphoma with a median survival time of less than 12 months and with a remarkable geographical prevalence in Asia and South America [1-2]. No standard effective treatment currently exists, as ENKTL is refractory to chemotherapy and is associated with a high rate of therapeutic failure and poor prognosis [3-4]. Developing optimal approaches for the early identification of patients at high risk of progression or relapse is important in clinical management. The most common approaches include histopathological subtyping and use of the International Prognostic Index (IPI) [5] and the Korean Prognostic Index (KPI) [6]. Additionally, the immunophenotype and gene expression pattern can be prognostic factors. However, previous prognostic indicators based on the presented parameters have several drawbacks, including lack of consideration of the lymphoma response to treatment and insufficiency for accurately identifying patients with immunochemotherapy-refractory disease [7-8].  Currently, 18F-ﬂuorodeoxyglucose (FDG) positron emission tomography-computed tomography (PET/CT) plays an important role in staging, response monitoring and prognostication assessments of lymphoma, especially Hodgkin lymphoma (HL) and diffuse large B-cell lymphoma (DLBCL) [9-11]. As a commonly used semi-quantitative parameter, the maximum standardized uptake value (SUVmax) is a survival predictor that is associated with tumor aggressiveness [12-14]. Several recent studies have shown that quantitative metrics including metabolic tumor volume (MTV) and total lesion glycolysis (TLG) are reliable prognostic indicators with high sensitivity and reliability in DLBCL [9-10, 15]. Additionally, the five-point Deauville score (DS) has been recommended as a qualitative method for evaluating interim and end-of-treatment PET/CT results with good reproducibility and flexibility [16-17]. Although previous studies have evaluated the prognostic value of these PET/CT parameters for various types of lymphoma, similar studies of ENKTL have been limited by sample size. Moreover, previous findings remain controversial due to heterogenous patient enrollment, various imaging conditions, different cut-off values for survival predictions, and undefined treatment protocols. Therefore, the purpose of this meta-analysis was to evaluate the prognostic value of multiple PET/CT parameters including SUVmax, MTV, TLG and DS in ENKTL at three different time points: baseline, interim and end of treatment. |  |
| Objectives | 4 | To evaluate the prognostic value of multiple PET/CT parameters including SUVmax, MTV, TLG and DS in ENKTL at three different time points: baseline, interim and end of treatment, we reviewed 3 prospective studies and 6 retrospective studies. 18F-FDG PET/CT results were determined on the basis of including SUVmax, MTV, TLG and DS examined for their ability to predict progression-free survival (PFS) and overall survival (OS). |  |
| **METHODS** | | |  |
| Protocol and registration | 5 | No |  |
| Eligibility criteria | 6 | (i) prospective or retrospective studies that used at least one of three PET/CT scans, namely, B-PET/CT, I-PET/CT and E-PET/CT, to assess therapeutic responses and to predict ENKTL patient survival; (ii) studies that included more than 10 patients with histologically confirmed ENKTL; (iii) studies containing survival data from which the hazard ratio (HR) was extractable, reporting at least one form of survival data progression-free survival (PFS) or overall survival (OS), or providing sufficient data for indirect extraction; (iv) when the data were presented in more than one study, the studies with the most complete or most recently published data were included; (v) and studies written only in English |  |
| Information sources | 7 | The PubMed, EMBASE, Cochrane Library and Medline databases were searched for English language articles. Additionally, reference lists from the included studies were also searched. |  |
| Search | 8 | The electronic search strategy for PubMed was as follows: Search ((((((ENKTL AND English[lang])) OR (NK/T-cell lymphoma AND English[lang])) OR (Lymphoma, Extranodal NK-T-Cell AND English[lang])) AND English[lang])) AND (((((PET CT AND English[lang])) OR (Positron Emission Tomography AND English[lang])) OR (Positron Emission Tomography Computed Tomography AND English[lang])) AND English[lang]) Filters: English  The electronic search strategy for EMBASE was: (positron emission tomography computed tomography.mp. or PET/CT or positron emission tomography.mp. or fluorodeoxyglucose.mp. or FDG.mp.) and (extranodal natural killer T cell lymphoma or lymphoma, Extranodal NK-T-Cell or extranodal natural killer T-cell lymphoma, nasal type or ENKTL).mp. [mp=title, abstract, heading word, drug trade name, original title, device manufacturer, drug manufacturer, device trade name, keyword, floating subheading word] |  |
| Study selection | 9 | Eligibility assessment was performed independently in an unblinded standardized manner by 2 reviewers. Disagreements between reviewers were resolved by consensus. |  |
| Data collection process | 10 | We developed a data extraction sheet (based on the Cochrane Consumers and Communication Review Group's data extraction template), pilot-tested it on 9 included studies. Two review authors extracted the following data from included studies independently. Disagreements were resolved by discussion between the two review authors; if no agreement could be reached, it was planned a third author would decide. |  |
| Data items | 11 | Information was extracted from each included trial on: study characteristics, including title, author, and publication time; patient characteristics, including age, gender, Ann Arbor stage, IPI score, treatment regimen, and follow-up time; imaging techniques, including the imaging system, interval time between FDG administration and scanning, FDG dose, and imaging interpreters; and survival data, including PFS and OS with HRs with 95% confidence intervals (95% CIs) for each. |  |
| Risk of bias in individual studies | 12 | The quality of the studies was evaluated independently by two reviewers. The Quality in Prognosis Studies (QUIPS) tool [18] was used to estimate the quality of the included studies. Using this tool, the validity and bias of the studies of prognostic factors were evaluated for the following six domains: study participation, study attrition, prognostic factor measurement, outcome measurement, study confounding, and statistical analysis and reporting. Each domain was rated as having a high, moderate, or low risk of bias considering the prompting items. |  |
| Summary measures | 13 | PFS and OS with 95% confidence intervals (95% CIs) were the primary measure of treatment effect. |  |
| Synthesis of results | 14 | Statistical heterogeneity was measured using the chi-squared Q test and the I2 statistic. We considered heterogeneity to be present at P<0.05 or/and I2>50%. A fixed effects model was used for the meta-analysis when heterogeneity was not significant, and a random effects model was used if heterogeneity was significant. The analyses described above were conducted by Review Manager (RevMan, version 5.3; The Nordic Cochrane Center, The Cochrane Collaboration). |  |

Page 1 of 2

| **Section/topic** | **#** | **Checklist item** | **Reported on page #** |
| --- | --- | --- | --- |
| Risk of bias across studies | 15 | For each trial we plotted the effect by the inverse of its standard error. The symmetry of such ‘funnel plots’ was assessed both visually, and formally with Begg’s test and Egger’s test, to see if the effect decreased with increasing sample size. |  |
| Additional analyses | 16 | no |  |
| **RESULTS** | | |  |
| Study selection | 17 | A total of 9 studies were identified for inclusion in the review. The search of PubMed, EMBASE, Cochrane Library and Medline databases.The search strategy was used to screen 106 records for inclusion. After discarding duplicate and irrelevant articles by reading the titles and abstracts, we reviewed 71 studies in detail. Among these articles, 62 studies were excluded for the following reasons: a lack of original data, such as case reports, letters, conference proceedings, commentaries and reviews (n=29); a focus on other diseases or lymphoma types (n=16); an aim toward revealing the diagnostic or staging value of PET/CT in ENKTL (n=11); or insufficient data to calculate PFS, OS and the HRs for each (n=6). |  |
| Study characteristics | 18 | Methods:  The principal characteristics of the nine studies. All studies, including three prospective studies and six retrospective studies, were published between 2013 and 2017 and were performed at Asian institutions. The median follow-up time ranged from 19 to 45 months. Five studies focused on the prognostic value of B-PET/CT, three studies addressed the prognostic value of I-PET/CT performed during the 4 weeks after initial treatment, and three studies revealed the prognostic value of E-PET/CT performed after the final treatment with an interval of 3-4 weeks.  Participants:  The included studies involved 535 participants. The main inclusion criteria entailed patients with untreated ENKTL, histologically confirmed in accordance with the WHO classification and (ii) patients who underwent three PET/CT scans [the pretreatment (baseline), interim, and post-therapy scans].  Outcomes:  the baseline, interim, and end of treatment PET/CT can predict survival in ENKTL patients. |  |
| Risk of bias within studies | 19 | The methodologic quality of the nine studies was assessed using the QUIPS tool. Generally, the included studies were of moderate quality. In the domain of prognostic factor measurement, there was a moderate risk of bias in two studies (24, 29) because they did not reveal detailed information about the imaging acquisition. All studies were judged to be at moderate risk of bias in the domain of outcome measurement because they did not report whether histological biopsy was used to confirm treatment failure or relapse. Due to clinical limitations associated with this disease, a biopsy of every lesion suspected of recurrence or progression is impossible. Finally, there was a moderate risk of bias due to study confounding in six studies (22, 24, 26-29) because these studies included patients treated with heterogeneous treatment regimens. The quality assessment of the included studies according to the six QUIPS domains is displayed in Table 3. |  |
| Results of individual studies | 20 | Figure2 and Figure3 |  |
| Synthesis of results | 21 | Six studies [22-23, 27-29] revealed the value of B-PET/CT in evaluating the prognosis for ENKTL. SUVmax [22-23, 27-28], MTV [22, 27, 29] and TLG [22, 27] were used to predict PFS and OS. For SUVmax, the HRs for PFS and OS were 2.78 (95% CI 1.54-5.03, p=0.0007; χ2=3.81, P=0.28, I2=21%) (Fig 2A) and 4.78 (95% CI 2.29-9.96, p<0.0001; χ2=0.31, P=0.96, I2=0%) (Fig 2B), respectively. Furthermore, there was an association between a high MTV value and poor PFS and OS with HRs of 3.61 (95% CI 1.96-6.65, p<0.0001; χ2=1.28, P=0.53, I2=0%) (Fig 2C) for PFS and 3.20 (95% CI 1.55-6.60, p=0.002; χ2=2.98, P=0.22, I2=33%) (Fig 2D) for OS. For TLG, the HRs for PFS and OS were 5.62 (95% CI 1.94-16.33, p=0.001; χ2=0.12, P=0.73, I2=0%) (Fig 2E) and 7.76 (95% CI 1.79-33.58, p=0.006; χ2=0.02, P=0.89, I2=0%) (Fig 2F), respectively. Taken together, the SUVmax, MTV and TLG of B-PET/CT were significant predictors for PFS and OS in ENKTL patients.  Regarding the I-PET/CT parameters, three studies [22, 24-25] using DS were available, with HRs for PFS and OS of 5.15 (95% CI 2.71-9.80, p<0.00001; χ2=1.13, P=0.57, I2=0%) (Fig 3A) and 5.80 (95% CI 2.28-14.73, p=0.0002; χ2=1.58, P=0.45, I2=0%) (Fig 3B), respectively, indicating a significant association between a positive DS in I-PET/CT and poor PFS and OS. Three studies [21-22, 26] were available regarding the DS on E-PET/CT, with HRs for PFS and OS of 3.65 (95% CI 2.13-6.26, p<0.00001; χ2=0.02, P=0.99, I2=0%) (Fig 3C) and 3.32 (95% CI 1.79-6.15, p=0.0001; χ2=0.38, P=0.83, I2=0%) (Fig 3D), respectively, suggesting that a positive DS in E-PET/CT predicted worse survival. |  |
| Risk of bias across studies | 22 | All pooled data were statistically homogeneous. In evaluating publication bias, the results of Egger’s test and Begg's test were both insignificant (p>0.05), indicating no publication bias for the HRs. |  |
| Additional analysis | 23 | no |  |
| **DISCUSSION** | | |  |
| Summary of evidence | 24 | As a rare cancer type with a relatively high incidence in Asia and South America, ENKTL is diagnostically considered in significant proportions of patients undergoing disease progression or relapse after a good initial treatment response, especially when lesions are disseminated [7]. Zhou X et al. [30] performed a meta-analysis and systematic review to assess the role of 18F-FDG-PET/CT in the diagnosis and staging of NK/T-cell lymphoma in 135 patients from 6 studies, and results indicated that PET/CT represented a valuable diagnostic and staging tool for ENKTL. Further research is necessary to identify which prognostic indicators are the most suitable and reliable in the management of ENKTL patients and to determine how to optimize the use of prognostic indicators to identify individuals with poor prognoses. The current meta-analysis assessed the prognostic value of PET/CT at three different time points with four parameters comprising SUVmax, MTV, TLG, and DS in ENKTL. Based on a statistical analysis of 535 ENKTL patients, our meta-analysis demonstrated that SUVmax, MTV, TLG of B-PET/CT, DS on I-PET/CT and DS on E-PET/CT were predictors for PFS and OS in ENKTL patients, indicating that PET/CT can be used as a metabolic imaging approach to monitor treatment responses and to assess the prognosis of ENKTL patients in clinical practice.  Several studies have shown that baseline SUVmax can be a predictor of the treatment response and survival rate in ENKTL, which is consistent with our meta-analysis results [31-32]. Chang et al. [22] reported that a high SUVmax was an independent predictor of OS but not PFS. Conversely, Kim et al. [27] reported that a high B-PET SUVmax was a significant predictor of PFS but not OS. Whereas in the study of Kim et al. [27], the sample size was relatively small, and patients received CHOP/CHOP-like or EPOCH regimens, which may have led to disputable results regarding prognosis. Due to multidrug resistance in ENKTL, anthracycline-based chemotherapy (e.g., CHOP or CHOP-like regimens) is ineffective and has been replaced by an array of more effective non-anthracycline regimens [7].  For B-PET/CT, TLG tends to be superior to SUVmax and MTV in predicting survival. The reason might be that TLG is an ideal metabolic parameter that combines the SUV mean and MTV to combine assessments of tumor volume and metabolism. Our results are consistent with previous studies that showed the prognostic value of the PET/CT volume parameters for revealing the whole body metabolic tumor burden. Based on their cohort study of 103 DLBCL patients, Ceriani et al. [10] concluded that TLG was the most powerful predictor on baseline PET/CT. Mikhaeel et al. [15] found that the baseline PET MTV and TLG were the most prognostic quantitative measures and were superior to IPI and DS. In their study, Zhou et al. [9] found that tumor metabolic volume parameters were helpful in the management of DLBCL and that TLG was an especially striking predictor. Moreover, Chang et al. [22] reported that the negative predictive values (NPVs) of baseline SUVmax, MTV and TLG for PFS and OS were higher than the positive predictive values (PPVs) in 47 ENKTL patients. Notably, the NPVs of TLG in predicting PFS and OS were 90.5% and 95.2%, respectively. Thus, negative results may have a stronger relationship with good PFS and OS than positive results have with poor PFS and OS.  However, our meta-analysis revealed that MTV was not superior to SUVmax regarding the combined HRs of both PFS and OS, and several reasons may explain this discrepancy. First, the three included studies [22, 27, 29] regarding MTV had too few patients to be conclusive. Second, MTV represents the size of tumor tissues that exhibit active 18F-FDG uptake, the MTV measurements are not reliable, and reproducibility, especially for multiple, disseminated, and extensive lesions, is poor [33]. Moreover, a standard method for estimating MTV thresholds has not been defined [34-36]. Chang et al. [22] estimated an MTV threshold based on 40% of the SUVmax. Song et al. [29] and Kim et al. [27] used a fixed threshold with SUVmax values of 2.5 and 3.0, respectively. A fixed threshold may lack reproducibility due to the variability caused by biological and technological factors, whereas using a proportion of the SUVmax as a threshold may lead to misestimation of the calculated tumor volume in cases of heterogeneous or low uptake. Kanoun et al. [37] evaluated the impact of the differences in methodological calculations on MTV values in HL and found that MTV values were significantly affected by calculation methods; however, different MTV values were associated with prognosis. Several studies have reported that the adoption of an individualized thresholds based on the liver background can reduce the impact of different scanning techniques and patient factors in DLBCL and solid tumors [9, 38]. Thus, a standard measuring method for MTV is needed for more accurate assessments of ENKTL patients.  Additionally, our study suggests that positive DS on I-PET/CT has a significant association with reduced PFS and OS in ENKTL, and the results of our analysis based on DS on E-PET/CT are consistent with those of previous studies. In a study of 24 patients with newly diagnosed or relapsed histologically confirmed ENKTL, Khong et al. [32] concluded that mid-treatment PET/CT was a valuable tool for early treatment response assessments. In a cohort of 88 T/NK-cell lymphoma patients including 26 ENKTL patients, both interim and post-therapy PET/CT scans were independent predictors of PFS and OS [39]. I-PET/CT presumably plays a critical role in two aspects of clinical management of lymphoma by allowing evaluation of the therapeutic response and prediction of prognosis. Due to the rarity of ENKTL and lack of a current unified treatment strategy for ENKTL, data addressing issues such as whether altering therapy based on I-PET/CT results can improve patient survival and whether DS as a metric of I-PET/CT can accurately distinguish high-risk patients from low-risk patients in ENKTL are lacking. Thus, a conclusion on the practical usefulness of I-PET/CT in ENKTL cannot be clearly drawn.  Previous studies have confirmed that the DS, where values of 1-3 are defined as negative results and 4-5 are defined as positive results on I-PET/CT, can better predict PFS and OS, with good concordance among reviewers and inter-reader reliability in HL and NHL [40-42]. Jiang et al. [25] evaluated the prognostic value of I-PET/CT using the DS and found that NPVs were better than PPVs in predicting PFS and OS, with NPVs of 87.5% and 96.9%, respectively, and PPVs of 60.7% and 39.3%, respectively. The authors concluded that negative results may have a stronger relationship with good PFS and OS than positive results have with poor PFS and OS. However, Lim et al. [24] found that DS associated with EBV DNA has significant prognostic value in ENKTL patients who undergo autologous stem cell transplantation and that defining values of 3-5 as positive results can better differentiate survival. These data were similar to a study by Kim et al. [26], in which a new risk stratification method was suggested based on DS and EBV DNA at the end of treatment. Khong et al. [32] revealed that scores of 1-2 at the end of treatment may represent a complete metabolic response; the scores are equal to an evaluation using IHPC and may be more appropriate at the end of treatment to increase the NPV of PET. The Lugano classification suggests that a score of 3 generally indicates a good prognosis with standard treatment, especially at the interim scan. However, if de-escalation is investigated, it may be preferable to consider a score of 3 as an inadequate response to avoid undertreatment [17]. Therefore, a score of 3 should be carefully interpreted depending on the timing of the assessment, the clinical context, and the treatment choice. Moreover, the risk of inter-observer subjectivity in the interpretation has not been eliminated, especially for small or slightly enhanced lesions in the background with changes in physiological uptake [43]. In one report, 22% of the cases had to be discussed due to discrepant DS assessments between the two independent interpreters [44]. The combined assessment is presumably a better predictor of survival outcomes than the single-parameter assessment, thus optimizing this assessment metric and reducing inter-observer differences are important. Cai QQ et al. [45] proposed a prognostic model including fasting blood glucose, total protein, and KPI for ENKTL, and the results demonstrated that the model could distribute patients into different risk groups with better prognostic discrimination than KPI alone. Lee J et al. [46] used a prognostic model that included B symptoms, stage, lactate dehydrogenase (LDH) level, and regional lymph nodes. Kim et al. [47] created new prognostic models, PINK and PINK-E, that were performed after patients received non-anthracycline-based treatment and considered age, stage, distant lymph-node involvement, non-nasal type disease, and Epstein-Barr virus DNA to guide risk-adapted treatment. According to Ya Jun Li et al. [48], the Glasgow Prognostic Score (GPS), a cumulative prognostic score based on CRP and albumin levels, is an independent predictor of survival outcomes in low-risk groups of patients with ENKTL and is superior to IPI, PIT, and KPI. Few studies have explored prognostic models that consider PET/CT scan results in ENKTL. Our meta-analysis shows the significant prognostic values of PET/CT in ENKTL and that the response assessment and survival prediction based on multiple PET/CT parameters and clinical characteristics should be considered in the management of ENKTL patients. |  |
| Limitations | 25 | The current meta-analysis has several limitations. First, only published English language articles were included, and all studies were from Asia. Second, the included studies enrolled relatively small numbers of subjects, and we analyzed a total of 535 ENKTL patients in this meta-analysis. Furthermore, the included studies were mostly retrospective, and the quality assessment was moderate. Registrations on Cochrane and PROSPERO are not available for this meta-analysis. These methodological study limitations contribute to deficiencies associated with the current study. Fourth, there were varying inclusion and exclusion criteria for patient enrollment among the included studies. According to Jiang et al. [21, 25], patients were excluded if they were diagnosed with primary cutaneous T/natural killer-cell lymphomas or anaplastic lymphoma kinase-positive anaplastic large cell lymphoma. In the cohorts of several included studies [21-22, 25], patients with central nervous system involvement were also excluded. Obviously, the treatment effect and prognosis may vary with histologic subtype or disease involvement. Finally, the minimum follow-up time was 19 months in the included studies. However, most relapses take longer to occur. If a longer follow-up time was used, the disease relapse rate would be higher, and the prognostic performance would be different. |  |
| Conclusions | 26 | The current evidence demonstrates that SUVmax, MTV, TLG of B-PET/CT, DS on I-PET/CT and DS on E-PET/CT may be significant prognostic indicators for PFS and OS in ENKTL. Moreover, for B-PET/CT, TLG tends to be superior to SUVmax and MTV in predicting the survival of ENKTL patients. Taken together, PET/CT assessments with multiple parameters may have better prognostic performance for determining ENKTL progression in patients. However, future large-scale prospective studies are needed to confirm the prognostic value of the multiple PET/CT parameters in ENKTL. |  |
| **FUNDING** | | |  |
| Funding | 27 | no |  |

*From:*  Moher D, Liberati A, Tetzlaff J, Altman DG, The PRISMA Group (2009). Preferred Reporting Items for Systematic Reviews and Meta-Analyses: The PRISMA Statement. PLoS Med 6(7): e1000097. doi:10.1371/journal.pmed1000097

For more information, visit: **www.prisma-statement.org**.

Page 2 of 2
