# Supplementary material for: Prognostic value of baseline, interim and end-of-treatment 18F-FDG PET/CT parameters in extranodal natural killer/T-cell lymphoma: A meta-analysis
Source: PLoS One. 2018 Mar 20;13(3):e0194435. doi: 10.1371/journal.pone.0194435 (PMC5860776; doi:10.1371/journal.pone.0194435)
Supplement: S1 Appendix — (DOCX) [file pone.0194435.s002.docx]

The electronic search strategy for PubMed was as follows:

#1 Search ENKTL Filters: English 163

#2 Search Lymphoma, Extranodal NK-T-Cell Filters: English 950

#3 Search NK/T-cell lymphoma Filters: English 1077

#4 Search (((ENKTL AND English[lang])) OR (NK/T-cell lymphoma AND English[lang])) OR (Lymphoma, Extranodal NK-T-Cell AND English[lang]) Filters: English 1324

#5 Search PET CT Filters: English 47259

#6 Search Positron Emission Tomography Filters: English 71722

#7 Search Positron Emission Tomography Computed Tomography Filters: English 39200

#8 Search (((PET CT AND English[lang])) OR (Positron Emission Tomography AND English[lang])) OR (Positron Emission Tomography Computed Tomography AND English[lang]) Filters: English 77132

#9 Search ((((((ENKTL AND English[lang])) OR (NK/T-cell lymphoma AND English[lang])) OR (Lymphoma, Extranodal NK-T-Cell AND English[lang])) AND English[lang])) AND (((((PET CT AND English[lang])) OR (Positron Emission Tomography AND English[lang])) OR (Positron Emission Tomography Computed Tomography AND English[lang])) AND English[lang]) Filters: English 73

The electronic search strategy for EMBASE was:

#1 positron emission tomography/computed tomography OR PET/CT OR positron emission tomography-computed tomography OR PET-CT OR fuorodeoxyglucose OR FDG OR 18F-FDG OR 18FDG OR FDG-F18 {including related terms} 10018

#2 (extranodal natural killer T cell lymphoma or lymphoma, Extranodal NK-T-Cell or extranodal natural killer T-cell lymphoma, nasal type or ENKTL).mp. [mp=title, abstract, heading word, drug trade name, original title, device manufacturer, drug manufacturer, device trade name, keyword, floating subheading word] 531

#3 (positron emission tomography computed tomography.mp. or PET/CT or positron emission tomography.mp. or fluorodeoxyglucose.mp. or FDG.mp.) and (extranodal natural killer T cell lymphoma or lymphoma, Extranodal NK-T-Cell or extranodal natural killer T-cell lymphoma, nasal type or ENKTL).mp. [mp=title, abstract, heading word, drug trade name, original title, device manufacturer, drug manufacturer, device trade name, keyword, floating subheading word] 74

#4 limit 2 to English language 70
