# Supplementary material for: Expression of Concern: Prognostic value of baseline, interim and end-of-treatment 18F-FDG PET/CT parameters in extranodal natural killer/T-cell lymphoma: A meta-analysis
Source: PLoS One. 2023 Oct 11;18(10):e0292986. doi: 10.1371/journal.pone.0292986 (PMC10566707; doi:10.1371/journal.pone.0292986)
Supplement: S2 File — (DOCX) [file pone.0292986.s002.docx]

**Primary Data Used for Analysis**

The following information is detailed in this file: study identifiers (i.e., first author and year), effect sizes and their variances (in log scale), and all required grouping variables for the analyses.

We included nine studies [21-29] and analyzed the prognostic value of FDG PET/CT parameters in extranodal NKT lymphoma. We grouped the analysis by the three time points at which FDG PET/CT parameters were collected.

- Group 1 – **Baseline FDG PET/CT**. Three parameters were reported (SUVmax, MTV, and TLG) and we further performed subgroup analysis for each parameter. Primary data were shown as **Table 1** in this file and results were presented as Figure 2 in the published article.
- Group 2 – **Interim FDG PET/CT**. Only one parameter was reported (Deauville 5-point scale). Primary data were shown as **Table 2** in this file and results were presented as Figure 3 (A and B) in the published article.
- Group 3 – **End-of-treatment FDG PET/CT**. Only one parameter was reported (Deauville 5-point scale). Primary data were shown as **Table 3** in this file and results were presented as Figure 3 (C and D) in the published article.

**Table 1. Primary data used for analyzing the prognostic value of baseline PET/CT parameters (Figure 2 in published article)**

| **Study identifiers** | **Progression-free Survival** | | | **Overall Survival** | | |
| --- | --- | --- | --- | --- | --- | --- |
|  | **HR*** | **95 % CI** | **P value** | **HR*** | **95 % CI** | **P value** |
| **SUV_max_** |  |  |  |  |  |  |
| Chang et al, 2017 [22] | 1.239 | 0.442–3.472 | 0.683 | 6.671 | 1.413–31.501 | 0.017 |
| Liang et al, 2016 [23] | 3.67 | 1.19–11.29 | 0.023 | 4.51 | 1.02–19.91 | 0.047 |
| Kim et al, 2013 [27] | 6.18 | 1.1–33.4 | 0.036 | 5.57 | 0.6–45.3 | 0.111 |
| Bai et al, 2013 [28] | 3.931 | 1.244–12.425 | 0.004 | 3.931 | 1.244–12.425 | 0.020 |
| **MTV** |  |  |  |  |  |  |
| Chang et al, 2017 [22] | 2.313 | 0.827–6.472 | 0.110 | 1.209 | 0.303–4.824 | 0.788 |
| Kim et al, 2013 [27] | 5.96 | 1.4–25.3 | 0.016 | 8.37 | 1.0–67.9 | 0.048 |
| Song et al, 2013 [29] | 4.17 | 1.714–10.147 | 0.002 | 4.102 | 1.617–10.408 | 0.003 |
| **TLG** |  |  |  |  |  |  |
| Chang et al, 2017 [22] | 6.94 | 1.413–34.12 | 0.017 | 8.632 | 1.090–68.336 | 0.041 |
| Kim et al, 2013 [27] | 4.74 | 1.1–19.9 | 0.035 | 6.97 | 0.8–55.5 | 0.068 |

SUV_max_ , maximum standardized uptake value; MTV, metabolic tumor volume;TLG, total lesion glycolysis

*When extracting the HR, we attempted to use the HR from the multivariate Cox analysis reported within the text of the report. When a study did not provide this information, we used the HR from the univariate Cox analysis instead. If the trial did not provide the HR directly, we attempted to indirectly extract it from the Kaplan-Meier survival curve as previously described.

**Table 2. Primary data used for analyzing the prognostic value of interim PET/CT parameters (Figure 3 [A and B] in published article)**

| **Study identifiers** | **Progression-free Survival** | | | **Overall Survival** | | |
| --- | --- | --- | --- | --- | --- | --- |
|  | **HR*** | **95 % CI** | **P value** | **HR*** | **95 % CI** | **P value** |
| Chang et al, 2017 [22] | 5.355 | 1.646–17.424 | 0.005 | 5.596 | 1.159–27.015 | 0.032 |
| Lim et al, 2016 [24] | 3.39 | 1.06–9.85 | 0.039 | 3.56 | 0.87–14.47 | 0.076 |
| Jiang et al, 2015 [25] | 7.787 | 2.580–23.504 | <0.001 | 17.494 | 2.247–136.217 | <0.001 |

﻿﻿ DS, Deauville 5-point scale

*HR was extracted using the same method as described in table 1.

**Table 3. Primary data used for analyzing the prognostic value of end-of-treatment PET/CT parameters (Figure 3 [C and D] in published article)**

| **Study identifiers** | **Progression-free Survival** | | | **Overall Survival** | | |
| --- | --- | --- | --- | --- | --- | --- |
|  | **HR*** | **95 % CI** | **P value** | **HR*** | **95 % CI** | **P value** |
| Jiang et al, 2017 [21] | 3.542 | 1.135–11.056 | 0.029 | 3.535 | 1.135–11.012 | 0.029 |
| Chang et al, 2017 [22] | 3.926 | 1.189–12.963 | 0.025 | 4.74 | 1.127–19.939 | 0.034 |
| Kim et al, 2015 [26] | 3.607 | 1.772–7.341 | <0.001 | 2·827 | 1.205–6.632 | 0.017 |

DS, Deauville 5-point scale

*HR was extracted using the same method as described in table 1.

**Reference (Reference number is consistent with the published article)**

21. Jiang C, Liu J, Li L, Kosik RO, Su M, Zou L, et al. Predictive approaches for post-therapy PET/CT in patients with extranodal natural killer/T-cell lymphoma: a retrospective study. Nuclear medicine commu- nications. 2017; 38(11):937–47. https://doi.org/10.1097/MNM.0000000000000731 PMID: 28858180

22. Chang Y, Fu X, Sun Z, Xie X, Wang R, Li Z, et al. Utility of baseline, interim and end-of-treatment 18F- FDG PET/CT in extranodal natural killer/T-cell lymphoma patients treated with L-asparaginase/pegas- pargase. Scientific reports. 2017; 7:41057. https://doi.org/10.1038/srep41057 PMID: 28117395

23. Liang JH, Ding CY, Gale RP, et al. Prognostic value of whole-body SUVmax of nodal and extra- nodal lesions detected by 18F-FDG PET/CT in extra-nodal NK/T-cell lymphoma. Oncotarget. 2017; 8:1737–1743. https://doi.org/10.18632/oncotarget.13873 PMID: 27974685

24. Lim SH, Hyun SH, Kim HS, Lee JY, Yoo KH, Jung KS, et al. Prognostic relevance of pretransplant Deauville score on PET-CT and presence of EBV DNA in patients who underwent autologous stem cell transplantation for ENKTL. Bone marrow transplantation. 2016; 51(6):807–12. https://doi.org/10.1038/ bmt.2016.6 PMID: 26855154

25. Jiang C, Su M, Kosik RO, Zou L, Jiang M, Tian R. The Deauville 5-Point Scale Improves the Prognostic Value of Interim FDG PET/CT in Extranodal Natural Killer/T-Cell Lymphoma. Clinical nuclear medicine. 2015; 40(10):767–73. https://doi.org/10.1097/RLU.0000000000000892 PMID: 26164182

26. Kim SJ, Choi JY, Hyun SH, Ki CS, Oh D, Ahn YC, et al. Risk stratification on the basis of Deauville score on PET-CT and the presence of Epstein-Barr virus DNA after completion of primary treatment for extranodal natural killer/T-cell lymphoma, nasal type: a multicentre, retrospective analysis. The Lancet Haematology. 2015; 2(2):e66–74. https://doi.org/10.1016/S2352-3026(15)00002-2 PMID: 26687611

﻿27. Kim C-Y, Hong CM, Kim D-H, Son SH, Jeong SY, Lee S-W, et al. Prognostic value of whole-body meta- bolic tumour volume and total lesion glycolysis measured on 18F-FDG PET/CT in patients with extrano- dal NK/T-cell lymphoma. European journal of nuclear medicine and molecular imaging. 2013; 40 (9):1321–9. https://doi.org/10.1007/s00259-013-2443-6 PMID: 23674211

28. Bai B, Huang HQ, Cai QC, Fan W, Wang XX, Zhang X, et al. Predictive value of pretreatment posi- tron emission tomography/computed tomography in patients with newly diagnosed extranodal natu- ral killer/T-cell lymphoma. Medical oncology. 2013; 30(1):339. https://doi.org/10.1007/s12032-012- 0339-0 PMID: 23329306

29. Song MK, Chung JS, Shin HJ, Moon JH, Ahn JS, Lee HS, et al. Clinical value of metabolic tumor volume by PET/CT in extranodal natural killer/T cell lymphoma. Leukemia research. 2013; 37(1):58–63. https:// doi.org/10.1016/j.leukres.2012.09.011 PMID: 23040533
